# Change in the local impedance and electrograms recorded by a micro‐electrode tip catheter during initial atrial fibrillation ablation

**DOI:** 10.1002/joa3.12535

**Published:** 2021-04-07

**Authors:** Kenji Hashimoto, Ippei Tsuzuki, Yuta Seki, Susumu Ibe, Terumasa Yamashita, Hiroshi Miyama, Taishi Fujisawa, Yoshinori Katsumata, Takehiro Kimura, Keiichi Fukuda, Seiji Takatsuki

**Affiliations:** ^1^ Department of Cardiology Keio University School of Medicine Tokyo Japan

**Keywords:** atrial fibrillation, catheter ablation, impedance

## Abstract

**Background:**

A novel measurement of the local impedance (LI) and electrograms recorded from micro‐electrodes on catheter tip has been developed. However, the data during pulmonary vein (PV) ablation is not sufficient. We aimed to investigate the utility of this measurement during initial atrial fibrillation (AF) ablation.

**Methods:**

We investigated 111 representative radiofrequency applications in 7 AF patients without a history of prior ablation (6 males, age 68 [65‐72] years, 2 persistent AF). The ablation strategy was PV isolation for paroxysmal AF and single ring box isolation for persistent AF, using MiFi catheter. The correlation of the generator impedance (GI) drop and LI drop after radiofrequency applications and the predictive value of the initial LI elevation before radiofrequency applications for LI drop were analyzed. Also, the LI and GI drop were investigated according to the location of RF applications.

**Results:**

The LI drop was higher than GI drop (23.7 [16.4‐35.7] and 9.0 [6.0‐12.0]; *P* < .01). There were correlations between the initial LI elevation and LI drop (*R* ^2^ = 0.466, *P* < .01) and between the LI and GI drop (*R* ^2^ = 0.263, *P* < .01). The LI drops significantly differed according to the different anatomical localizations by the Kruskal–Wallis test, although the GI drops did not differ (*P* < .01 and *P* = .49, respectively)

**Conclusion:**

LI drop was associated with initial LI elevation and was larger than GI drop. LI drop was different according to locations, although GI drop was not. These findings might indicate that LI drop would be a more sensitive marker for lesion formation than GI drop.

## Graphical abstract

Utility of the novel measurement of the local impedance during initial atrial fibrillation ablation was investigated. The local impedance drop during radiofrequency applications was higher than the generator impedance drop, both of which were positively correlated.

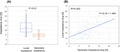

## 1 INTRODUCTION

An electrical pulmonary vein (PV) isolation by catheter ablation has been established as a curative treatment for paroxysmal atrial fibrillation (AF).^1^ Since most paroxysmal AF patients are able to be cured by PV isolation and recovery of PV conduction is one of the most frequent causes of AF recurrence, the durability of the PV isolation is essential.^2^, ^3^

Successful radiofrequency (RF) catheter ablation is dependent on the ability to deliver an effective lesion with good and stable tissue contact. Contact force (CF) between the catheter tip and myocardium has been identified as one of the most important determinants influencing the RF lesion size as well as the RF power and duration.^4^, ^5^, ^6^ Other than the CF, the Force‐Time Integral and Ablation Index have been shown to have a positive correlation with better patient outcomes.^4^, ^7^ However, such parameters represented factors on the side of delivering RF energy and did not imply the state of the tissue. On the other hand, the generator impedance (GI) between the catheter tip and cutaneous patch is widely known as an electrical index of lesion formation.^8^ The previous study suggested that increased tissue contact was associated with a larger GI drop during RF applications.^9^ However, the starting GI was not shown to be associated with the CF or lesion creation, and information on GI decreases is available only after starting an RF application. In addition, GI measurements can be affected not only by the degree of contact with the myocardium but also by other thoracic structures.

Recently, a novel ablation catheter (Intellanav MiFi OI catheter, Boston Scientific) has been developed, which consists of 3 equally spaced 1‐mm diameter micro‐electrode incorporated into the tip. This novel catheter provides ultra‐high‐density mapping by micro‐electrode bipolar recordings and incorporates a “DirectSense” algorithm to measure the local tissue impedance (LI) from the distal micro‐electrodes.^10^ The resistive load on the distal electrode of an ablation catheter is influenced by the percentage of surface area covered by myocardium. LI is highly sensitive to objects with dissimilar resistivity. LI is able to distinguish the degree of catheter–tissue contact, because healthy myocardium displays a larger resistivity compared with blood pool. The past studies reported that this catheter provided a high electrogram spatial resolution, which may aid in identifying critical isthmuses and gaps on a line of block, and that the LI drop measurements during RF applications give an indication of the tissue contact and subsequent effective lesions.^11^, ^12^, ^13^, ^14^ However, the data on this novel catheter during the initial AF ablation procedure, such as a PV isolation and single ring box isolation, have scarcely been reported. Therefore, the present report investigated the efficacy of the measurement of the local electrogram and LI with a novel MiFi catheter during the initial AF ablation procedure.

## 2 METHODS

### 2.1 Study population

The RF applications from 7 patients undergoing an initial catheter ablation of paroxysmal or persistent AF at Keio University Hospital were retrospectively analyzed. PV isolations were performed in 5 patients and single ring box isolation in 2. The study protocol was approved by the institutional review board committee at the hospital. All participants provided written informed consent for the procedures.

### 2.2 Catheter ablation procedure

The ablation procedure was performed under deep sedation with propofol and monitored with a Bispectral Index monitor (Aspect Medical Systems), maintaining the value within the range of 40‐60. An oral airway and facial mask for auto servo‐ventilation (ResMed) were provided to stabilize the respirations. Unfractionated heparin was administered before the transseptal punctures to maintain an activated clotting time of 300‐400 seconds for the duration of the procedure.

A multielectrode catheter was introduced from the femoral vein and placed in the right ventricle, recording the His bundle electrogram with the proximal electrodes. Another multielectrode catheter was introduced from the right jugular vein and placed in the coronary sinus, to record the right atrial and superior vena cava electrograms with the proximal electrodes, which also could be used for intracardiac electrical cardioversion. The IntellaTip MiFi, an open‐irrigated ablation catheter with 3 mini‐electrodes incorporated into the tip, and IntellaMap Orion (Boston Scientific), a 64‐mini‐electrode basket mapping catheter were inserted into left atrium from the right femoral vein. To access the left atrium, two septal punctures were performed. Guidance using the RHYTHMIA electroanatomic mapping system (Boston Scientific) was used during the procedures.

**FIGURE 1:**
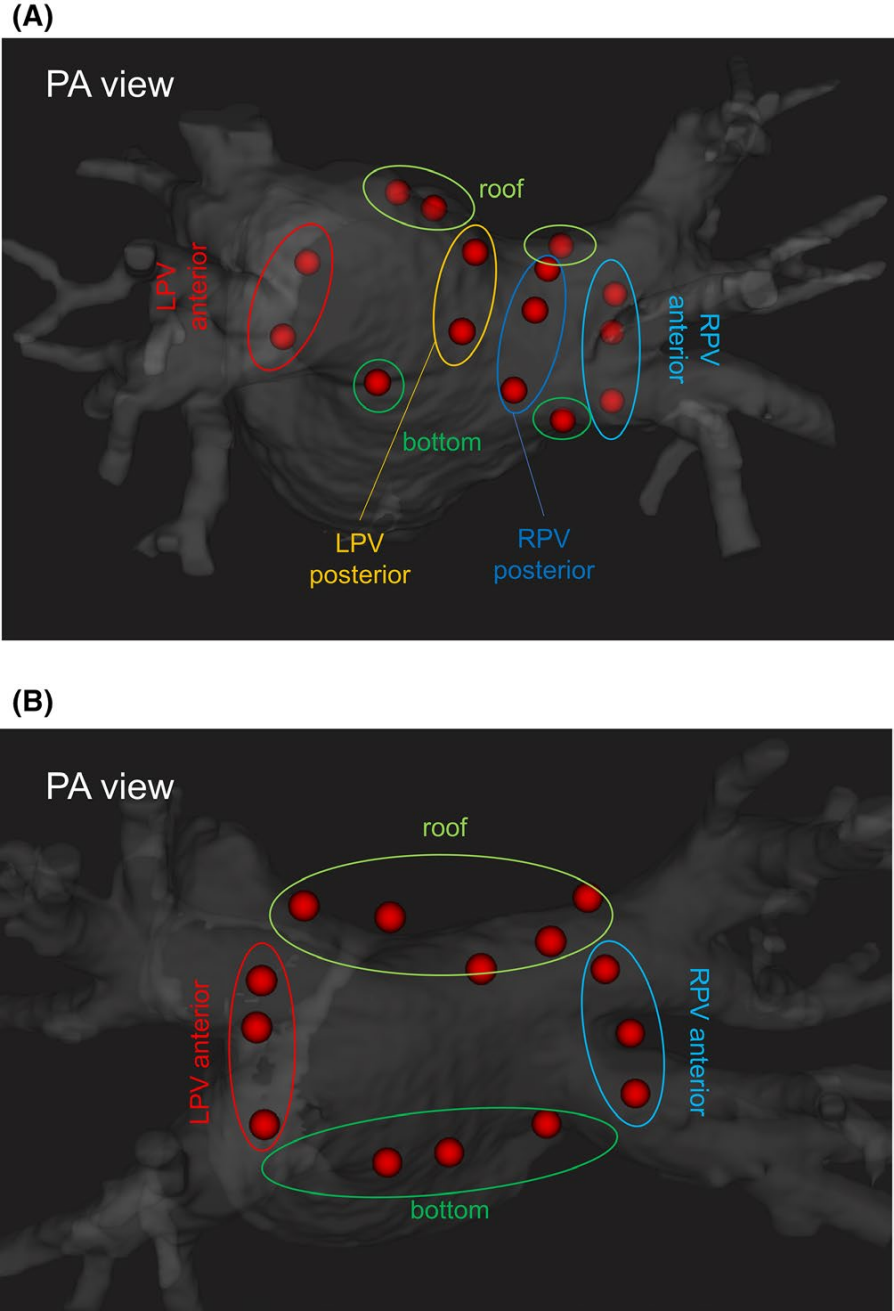
Radiofrequency applications assessed in the present study. We conducted the radiofrequency applications with an adequate distance from the prior ablation lesions at first, and then we added radiofrequency applications to anatomically fill the gaps. In order to exclude the influence of the adjacent ablation lesions, we only investigated the former radiofrequency applications, shown in this figure. A, For the pulmonary vein (PV) isolation, we investigated the radiofrequency applications on the PV anterior wall, posterior wall, roof portion, and bottom portion of each PV. B, For the box isolation, we investigated the radiofrequency applications on the left PV anterior wall, roof area, bottom area, and right PV anterior wall. LPV, left pulmonary vein; RPV, right pulmonary vein

The ablation strategies were an ipsilateral circumferential PV isolation for paroxysmal AF and box isolation including all four PVs and the left atrial posterior wall for persistent AF. RF energies were also delivered for non‐PV triggers or atrial tachycardias that were repeatedly induced by programmed electric stimulation with or without an infusion of isoproterenol. Further linear ablation or ablation of complex fractionated atrial electrograms was performed at the discretion of the operator. The ablation lesions were delivered using the same RF generator (Maestro 4000; Boston Scientific) in power‐controlled mode. The power delivery was set at 25 to 35 W depending on the left atrial location. The power settings were not changed during RF delivery and changes were made only between the RF applications. We conducted an RF application with an adequate distance from the prior ablation lesions where sharp local electrograms could be observed at first and then RF applications were added anatomically to fill the gaps. In order to exclude the influence of adjacent ablation lesions, we only investigated the former electrogram‐based RF applications from 6 distinct locations; left PV anterior and posterior walls, roof area, bottom area, and right PV anterior and posterior walls (Figure 1A,B).

The target LI drop for a single RF application was ≧20, and the RF application was forcibly stopped when the duration reached 30 seconds, LI drop reached 50 Ω, or esophageal temperature reached 39 degrees. The procedural endpoint was a bidirectional conduction block into and out of the PVs or box area. The surface and endocardial electrograms were continuously monitored and recorded for offline analysis (Bard Electrophysiology, Boston Scientific). The intracardiac electrogram was filtered from 30 to 250 Hz and then measured at a sweep speed of 100 mm/s.

### 2.3 Impedance assessment

The GI was measured from Maestro 4000 between the dome electrode and cutaneous patch. At the same time, the LI was measured from the micro‐electrodes of the MiFi ablation catheter by injecting a non‐stimulatory alternating current (5.0 μA, 14.5 kHz) between the tip electrode and proximal ring to create a local potential field. The amplitude of the potential field distortions, caused by contact with high resistivity myocardium, between each micro‐electrode and the distal ring was recorded. The potential measurement was converted into the impedance by dividing it by the injection current.^10^ The highest impedance among three impedance measurements by the micro‐electrodes was used as the LI. The reference LI was recorded in the blood pool of the left atrium prior to the initial RF application. The initial impedance just before the RF application and impedance drop after the RF application were measured both from the micro‐electrodes of the catheter and from the generator. The initial LI elevation was defined as the difference between the initial LI and reference LI. The impedance drop was defined as an impedance decrease during a RF application. The correlation of the GI and LI drop and predictive value of the initial LI elevation for the LI and GI drop were analyzed.

### 2.4 Electrogram amplitude assessment

The electrograms were recorded from the standard tip bipolar recordings from the dome to ring and the micro‐electrode bipolar recordings, just prior to the RF application and right after the RF application. For the electrogram from the micro‐electrode recording, the maximum value from the three micro‐electrode pair recordings was used. The electrogram amplitude recorded by the two different methods was compared during sinus rhythm and AF rhythm.

Also, in order to investigate the effectiveness of the micro‐electrode bipolar recordings in prior RF applicated regions, we assessed the electrogram amplitudes at the last five RF applications before completion of PV or box isolation in all cases. We compared the electrogram amplitudes using the standard tip bipolar recordings and micro‐electrode bipolar recordings in these ablation points before the RF applications started.

### 2.5 Statistical analysis

Continuous variables were presented as the median and interquartile range. Categorical variables were presented as numbers and percentages. Group differences were evaluated by a t test for continuous variables. Kruskal‐Wallis test was performed for an analysis of the LI and GI drop at the different anatomical locations. A linear regression analysis was calculated to determine the relationships between the GI and LI drop, as well as the initial LI elevation and impedance drop. All statistical calculations and analyses were performed using SPSS, version 24.0 software (IBM Corp.). Differences with *P* values <.05 were considered statistically significant.

## 3 RESULT

### 3.1 Study subject

The baseline characteristics of all 7 patients (6 males and 1 female) are listed in Table 1. Five patients had paroxysmal AF and two had persistent AF. The median and interquartile range age was 68 [65‐72] years of age, body mass index 23.9 [22.8‐25.4] kg/m^2^, CHA_2_DS_2_ Vasc score 1 [1‐2], and number of RF applications of interest per case 16 [15.5‐16.5]. No patients had structural heart disease. A total of 111 electrogram‐based RF applications was mainly assessed.

**TABLE 1:** Baseline characteristics of the study population

Patient no.	Age, year old	BMI, kg/m^2^	Gender (female)	Type (paroxysmal)	Procedure (PV isolation)	CHA2D2S Vasc score	Ablation points of interest
1	74	25.7	Female	Paroxysmal	PV isolation	2	13
2	65	20.9	Male	Paroxysmal	PV isolation	1	16
3	65	22.8	Male	Paroxysmal	PV isolation	1	15
4	73	23.9	Male	Paroxysmal	PV isolation	1	16
5	71	25.2	Male	Paroxysmal	PV isolation	2	18
6	68	30.4	Male	Persistent	box isolation	2	17
7	35	22.8	Male	Persistent	box isolation	0	16
Total	68 [65‐72]	23.9 [22.8‐25.4]	14.3%	71.4%	71.4%	1 [1‐2]	16 [15.5‐16.5]

Note

Data are the number of patients (%) or median [interquartile range].

Abbreviations: BMI, body mass index; CHA2DS2‐VASc, heart failure, hypertension, age 75 years (doubled), diabetes mellitus, stroke (doubled), vascular disease, age 65 to 74 years, and female; PV, pulmonary vein.

The ablation procedure was completed successfully in all cases. For the box isolation, two cases needed additional RF applications to completed the block lines after one round of RF applications. For the PV isolation, 9 PVs had completed block lines by one round of RF applications, and 1 PVs needed additional RF applications to complete the block line. There were no complications in any cases.

### 3.2 Impedance Assessment

**FIGURE 2:**
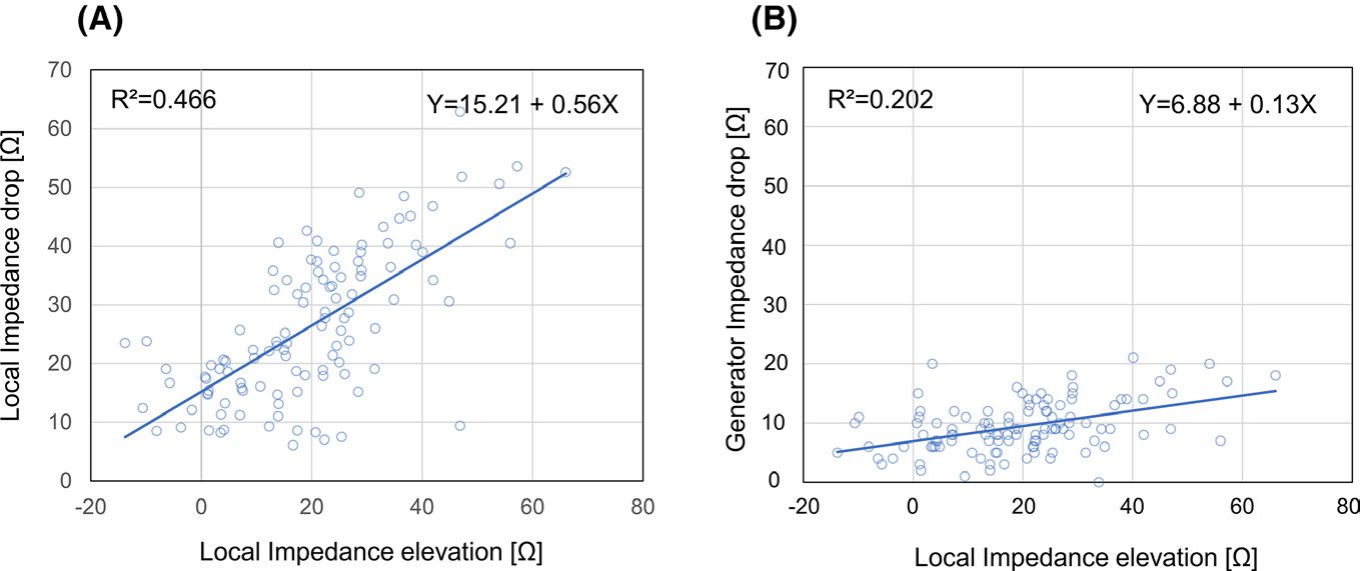
Scatterplot of the local impedance drop (A) and generator impedance (B) drop after the radiofrequency application as a function of the initial local impedance elevation. There was a correlation between the initial local impedance elevation and local impedance drop (*R* ^2 ^= 0.466, *P* < .01) and between the initial local impedance elevation and generator impedance (*R* ^2^ = 0.202, *P* < .01)

**FIGURE 3:**
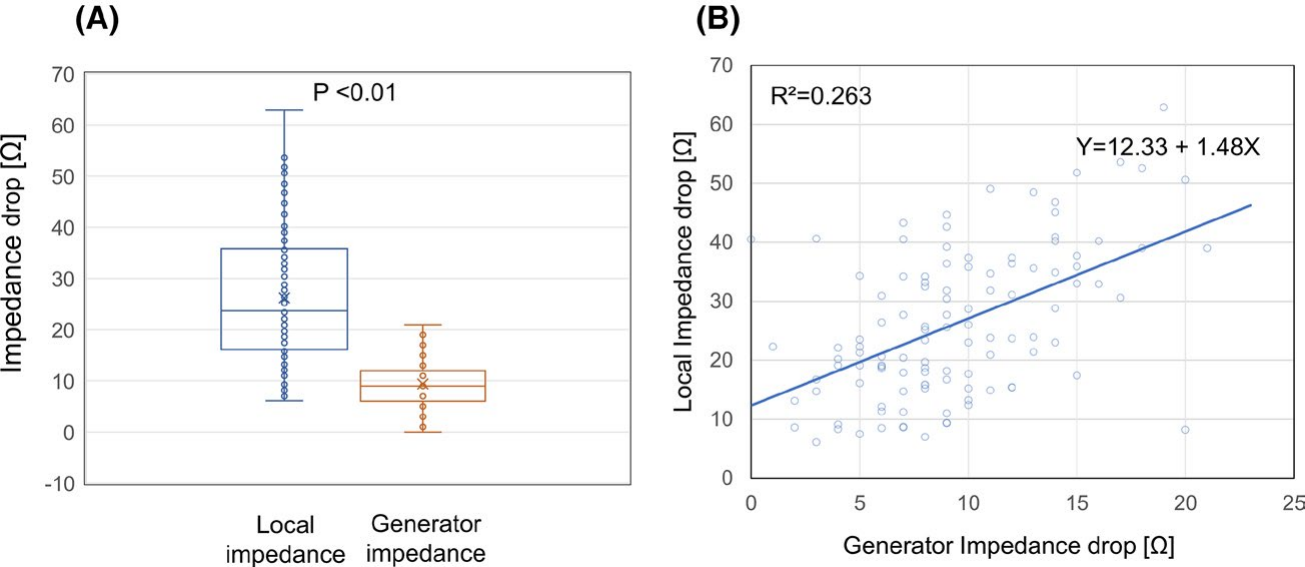
Comparison between the local impedance drop and generator impedance drop after the radiofrequency application. A, The median and interquartile local impedance and generator impedance drop after the radiofrequency application was 23.7 [16.4‐35.7] Ω and 9.0 [6.0‐12.0] Ω, respectively (*P* < .01). B, A scatterplot of the local impedance drop versus the generator impedance drop. There was a correlation between the local impedance drop and generator impedance drop after the radiofrequency application (*R* ^2^ = 0.263, *P* < .01)

**FIGURE 4:**
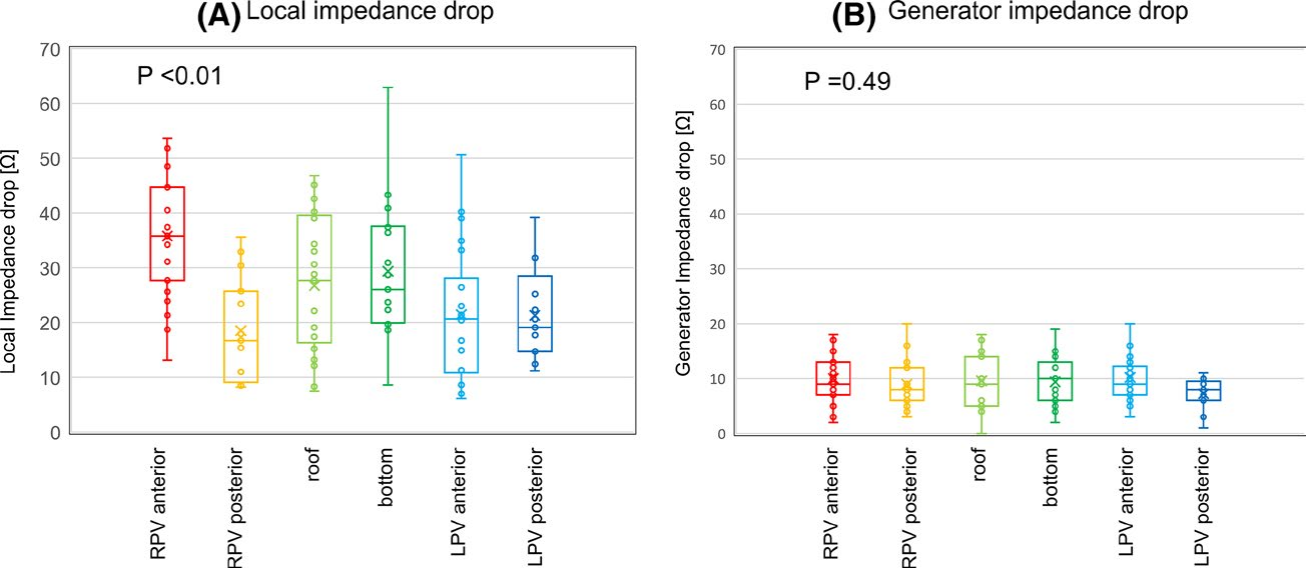
Local impedance drops (A) and generator impedance drops (B) among the different anatomical localizations. The local impedance (LI) and generator impedance (GI) drop was 35.8 [29.4‐42.7] Ω and 9.0 [7.5‐12.5] Ω for the right pulmonary vein (PV) anterior wall, 16.7 [10.1‐25.6] Ω and 8.0 [6.5‐10.5] Ω for the right PV posterior wall, 27.7 [17.4‐39.0] Ω and 9.0 [5.0‐14.0] Ω for the roof area, 26.0 [20.2‐37.4] Ω and 10.0 [6.0‐12.0] Ω for the bottom area, 20.7 [12.2‐25.8] Ω and 9.0 [7.3‐11.8] Ω for the left PV anterior wall, and 19.1 [14.7‐25.2] Ω and 8.0 [6.0‐9.0] Ω for the left PV posterior wall. The LI drops significantly differed according to the different anatomical localizations by the Kruskal–Wallis test, although the GI drops did not differ (*P* < .01 and *P* = .49, respectively).

The impedance data and electrogram amplitude data before and after the RF applications are summarized in Table 2. The median and interquartile duration of each RF application was 22.0 [19.0‐25.5] sec. The reference LI in seven cases was 102.0 [95.0‐105.0] Ω. The initial LI elevation was 19.9 [8.5‐27.9] Ω and was correlated with the LI drop and GI drop after the RF application (*Y* = 15.21 + 0.56*X*, *R* ^2 ^= 0.466, *P* < .01 and *Y* = 6.88 + 0.13*X*, *R* ^2^ = 0.202, *P* < .01; Figure 2). The LI drop and GI drop were 23.7 [16.4‐35.7] Ω and 9.0 [6.0‐12.0] Ω (*P* < .01), respectively, and there was a correlation between the LI drop and GI drop (*Y* = 12.33 + 1.48*X*, *R* ^2^ = 0.263, *P* < .01; Figure 3). As for the anatomical locations, the LI and GI drop was 35.8 [29.4‐42.7] Ω and 9.0 [7.5‐12.5] Ω for the right PV anterior wall, 16.7 [10.1‐25.6] Ω and 8.0 [6.5‐10.5] Ω for the right PV posterior wall, 27.7 [17.4‐39.0] Ω and 9.0 [5.0‐14.0] Ω for the roof area, 26.0 [20.2‐37.4] Ω and 10.0 [6.0‐12.0] Ω for the bottom area, 20.7 [12.2‐25.8] Ω and 9.0 [7.3‐11.8] Ω for the left PV anterior wall, and 19.1 [14.7‐25.2] Ω and 8.0 [6.0‐9.0] Ω for the left PV posterior wall. The LI drops significantly differed according to the different anatomical localizations by the Kruskal–Wallis test, although the GI drops did not differ (*P* < .01 and *P* = .49, respectively; Figure 4).

**TABLE 2:** Procedural parameters

	Patients (n = 7)
Local impedance	
Reference impedance, Ω	102.0 [95.0‐105.0]
Initial impedance, Ω	119.4 [108.8‐131.2]
Initial impedance elevation, Ω	19.9 [8.5‐27.9]
Impedance drop, Ω	23.7 [16.4‐35.7]
Generator impedance	
Initial impedance, Ω	87 [83‐93]
Impedance drop, Ω	9 [6‐12]
Initial MiFi amplitude, mV	1.0 [0.5‐1.9]
Post MiFi amplitude, mV	0.2 [0.1‐0.4]
Initial TIP amplitude, mV	0.6 [0.4‐1.1]
Post TIP amplitude, mV	0.2 [0.1‐0.3]
Duration of an RF application, sec	22.0 [19.0‐25.5]

Note

Data are the median [interquartile range].

The MiFi amplitude, micro‐electrode bipolar recordings, and maximum value from the three recordings of the micro‐electrode pairs were used. RF, radiofrequency; TIP amplitude, the standard tip bipolar recordings from the dome to ring.

### 3.3 Electrogram amplitude during sinus and atrial fibrillation rhythms

Among 111 RF applications assessed in the present study, 70 applications were conducted during sinus rhythm and 41 during an AF rhythm. During sinus rhythm, the median and interquartile electrogram amplitudes before the RF application was 0.91 [0.49‐1.60] mV with the standard tip bipolar recordings and 1.60 [0.80‐2.85] mV with the micro‐electrode bipolar recordings (*P* <.01). During the AF rhythm, the electrogram amplitudes before the RF applications was 0.43 [0.29‐0.55] mV with the standard tip bipolar recordings and 0.53 [0.30‐0.82] mV with the micro‐electrode bipolar recordings (*P* = .08). The electrogram amplitudes observed with the micro‐electrode bipolar recording during sinus rhythm were significantly higher than with the standard tip bipolar recording. Those with the micro‐electrode bipolar recordings during AF rhythm were tended to be higher, but not statistically significant.

**FIGURE 5:**
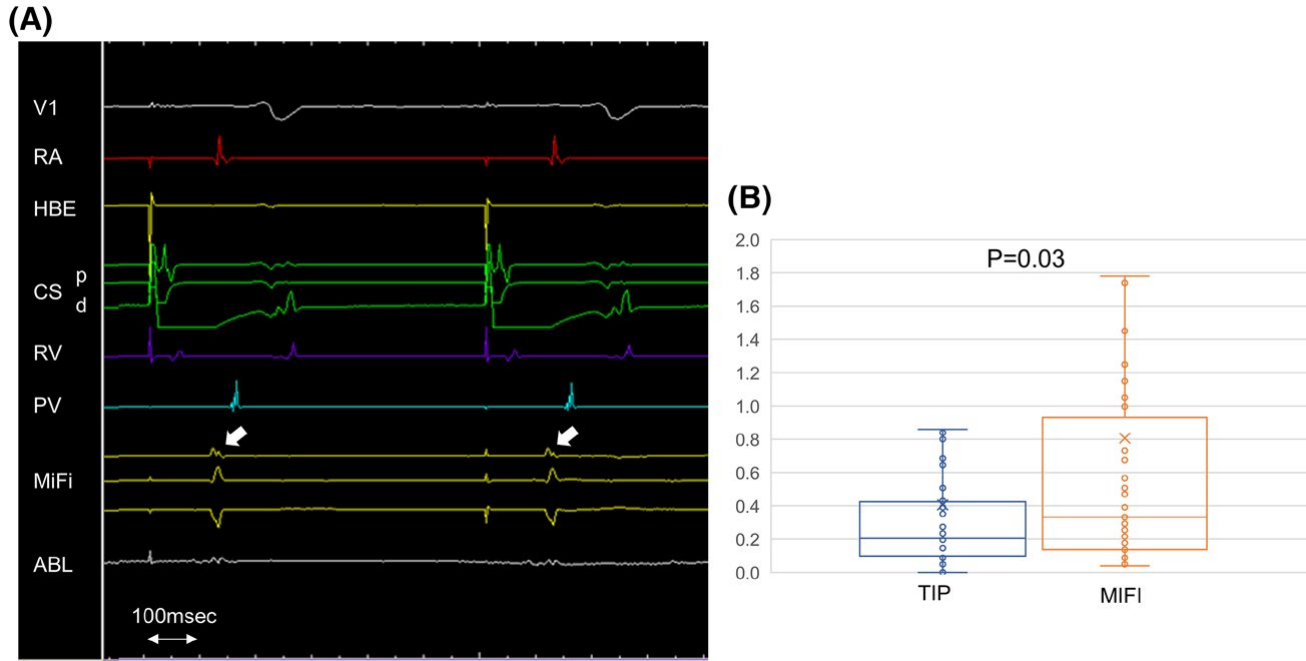
Comparison of the electrogram amplitudes between the standard tip bipolar recordings and micro‐electrode bipolar recordings in the last five radiofrequency applications before completing the isolations. A, Representative intracardiac electrogram. The electrogram amplitudes (white arrow) are higher and sharper in micro‐electrode bipolar recordings. B, The median and interquartile electrogram amplitudes were 0.21 [0.10‐0.42] mV for the standard tip bipolar recordings and 0.33 [0.14‐0.80] mV for the micro‐electrode bipolar recordings (*P* = .03). ABL, ablation catheter; CS‐d, distal coronary sinus; CS‐p, proximal coronary sinus; HBE, His bundle electrogram; MiFi, the electrogram recorded by micro‐electrode, PV, pulmonary vein; RA, right atrium

We also assessed the electrogram amplitudes at the last five RF application before completion of PV or box isolations in all cases, besides 111 electrogram‐based RF applications. In this investigation, we assessed 60 RF applications; 10 before box isolations and 50 before PV isolations. The median and interquartile electrogram amplitudes were 0.21 [0.10‐0.42] mV for the standard tip bipolar recordings and 0.33 [0.14‐0.80] mV for the micro‐electrode bipolar recordings (*P* = .03; Figure 5B). The representative intracardiac electrogram is shown in Figure 5A. The electrogram amplitudes were higher and sharper in micro‐electrode bipolar recordings, comparing to the standard tip bipolar recordings.

## 4 DISCUSSION

This was a retrospective observational study including patients who underwent an initial AF ablation (ipsilateral circumferential PV isolation or single ring box isolation). The major findings were as follows. First, the LI drop correlated with and was larger than the GI drop after the RF application. LI drop was different according to different locations, although GI drop was not. The LI drop was a more sensitive marker for the lesion formation created by an RF application. Second, the initial LI elevation before the RF application predicted an LI and GI drop. The initial LI elevation was useful to predict the lesion effectiveness before the RF application. Third, the electrogram amplitude recorded by the micro‐electrode bipolar recording using the MiFi catheter tended to be higher than that using the standard tip bipolar recording. The higher electrogram spatial resolution might help us to complete the PV isolation or single ring box isolation.

Several recent studies have investigated the impact of the LI on catheter ablation. The experimental study showed that the LI was valuable as a coupling indicator and that the additional use of the LI to the CF might improve the outcome of the ablation procedure.^10^, ^15^ In the clinical setting, Gunawardene et al analyzed that the LI in patients with recurrent AF or atrial tachycardia after a prior AF ablation, and Martin et al analyzed the LI in patients with a range of complex arrhythmias. Both studies showed that the value of the initial LI and LI drop after the RF application was a predictor of an effective lesion formation. However, during the PV isolation, which is the most common ablation procedure nowadays, the LI has been scarcely investigated. A few recent studies investigated the clinical utility of LI measurements during the PV isolation. Segreti et al reported that the LI drop after an RF application was higher than the GI drop and that the LI drop was an effective indicator of a successful lesion formation. Although the major findings were similar, the degree of the LI drop (14.8 Ω) and GI drop (3.7 Ω) differed from those of the present study.^13^ The differences in the patient background such as the BMI and contact between the catheter tip and myocardium might have been the reason for this difference. Masuda et al reported the utility of the LI drop (≧13.4 Ω) for creating sufficient ablation lesions assessed by the absence of a conduction gap during the PV isolation.^14^ Although that study population was similar to the present report, we delivered the RF applications located well away from the prior lesion at first and only assessed those RF applications in order to exclude the influence of the adjacent ablation lesions. Our study showed the effectiveness of the measurement of the initial LI elevation and LI drop in de novo ablation sites during the PV isolation. Furthermore, in the analysis of the anatomical localization, we found that the LI drop differed according to the localization in the left atrium, while the GI did not differ. This feature of LI might be a great advantage comparing to GI. For example, in the present study, LI drops in the right PV posterior wall were lower than in the right PV anterior wall. This might be because of the influence of the spine behind the ablation points. The presence of the spine behind the ablation points makes it difficult for the ablation catheter to cave in left atrial wall. Therefore, the percent of surface covered area in the distal electrode might be lower, resulting in lower impedance changes.

Another feature of the MiFi catheter is the electrogram recordings from the micro‐electrodes, which provide a higher electrogram spatial resolution and may aid in identifying the critical location during the ablation procedure.^11^ The present study showed that the electrogram amplitude recorded by the micro‐electrodes was larger than that by the standard tip bipolar recording from the dome to ring. By providing a high electrogram spatial resolution, this novel electrogram recording could aid in performing AF ablation, since we often need to conduct a PV isolation during an AF rhythm. In the additional assessment of the last five RF applications before completion of the PV or BOX isolations, the electrogram amplitudes in both standard tip bipolar recordings and micro‐electrode bipolar recordings were relatively low. Even in these low voltage regions with prior RF applications, the electrogram amplitudes by micro‐electrode bipolar recordings were significantly higher than the standard tip bipolar recordings, which suggested that the micro‐electrode bipolar recording could be useful in low voltage region such as diseased atria or atria with prior AF ablations.

## 5 LIMITATIONS

The present study had several limitations. First, this was a single‐center observational study; and therefore, the results were generally affected by multiple confounding factors. The limited sample size also influenced our analysis. Second, since no complications were recorded in the present study, the amount of the LI drop showing excessive contact was unknown. Third, it is quite helpful to represent an impedance curve in this investigation, however, only the initial and final LI values were saved in this version of RHYTHMIA system we used. Therefore, the tilts of LI changes were unknown. As the LI curves of all RF applications are saved in the new version of RHYTHMIA system, the LI curves should be investigated in the future study. Fourth, our study did not provide any information on the quality of the tissue contact and did not investigate the effectiveness of the RF applications, though the change in the amplitudes of the electrograms was assessed. Recently, a new catheter, which is able to measure both contact force and LI drop, began to be used in the clinical setting. The correlation between these two parameters should be investigated in the future study. Finally, our interpretations are only applicable to the de novo ablation sites, since the local impedance and local electrogram could have been affected by the prior adjacent RF application.

## 6 CONCLUSION

The LI drop was associated with the initial LI elevation and was larger than the GI drop, and LI drop was different according to different locations although GI drop was not. Our findings indicate that the LI drop would be a more sensitive marker for the lesion formation than the GI drop. The MiFi electrodes could record higher amplitudes of the local electrograms as compared to the conventional electrodes, which would be helpful for PV ablation.

## DISCLOSURE STATEMENT

### Conflict of interest

Authors declare no conflict of interests for this article.

### IRB approval number

20120275, IRB approval date: 2020/5/29.
